# Has the non-resection rate decreased during the last two decades among patients undergoing surgical exploration for pancreatic adenocarcinoma?

**DOI:** 10.1186/s12893-020-00835-3

**Published:** 2020-08-05

**Authors:** C. Mattevi, J. Garnier, U. Marchese, J. Ewald, M. Gilabert, F. Poizat, G. Piana, J. R. Delpero, O. Turrini

**Affiliations:** 1 Departement of Surgery, Institut Paoli-Calmettes, Marseille, France; 2 Departement of Oncology, Institut Paoli-Calmettes, Marseille, France; 3 Departement of Pathology, Institut Paoli-Calmettes, Marseille, France; 4 Departement of Radiology, Institut Paoli-Calmettes, Marseille, France; 5 Departement of Surgery, Aix-Marseille University, Institut Paoli-Calmettes, CRCM, 232 boulevard Sainte Marguerite, 13009 Marseille, France

**Keywords:** Pancreatic adenocarcinoma, CT, Liver MRI, Staging

## Abstract

**Purpose:**

To determine if improvement in imaging reduces the non-resection rate (NRR) among patients with pancreatic ductal adenocarcinoma (PDAC).

**Methods:**

From 2000 to 2019, 751 consecutive patients with PDAC were considered eligible for a intention-to-treat pancreatectomy and entered the operating room. In April 2011, our institution acquired a dual energy spectral computed tomography (CT) scanner and liver diffusion weighted magnetic resonance imaging (DW-MRI) was included in the imaging workup. We consequently considered 2 periods of inclusion: period #1 (February 2000–March 2011) and period #2 (April 2011–August 2019).

**Results:**

All patients underwent a preoperative CT scan with a median delay to surgery of 18 days. Liver DW-MRI was performed among 407 patients (54%). Median delay between CT and surgery decreased (21 days to 16 days, *P* < .01), and liver DW-MRI was significantly most prescribed during period #2 (14% vs 75%, *P* < .01). According to the intraoperative findings, the overall NRR was 24.5%, and remained stable over the two periods (25% vs 24%, respectively). While vascular invasion, liver metastasis, and carcinomatosis rates remained stable, para-aortic lymph nodes invasion rate (0.4% vs 4.6%; *P* < 0.001) significantly increased over the 2 periods. The mean size of the bigger extra pancreatic tumor significantly decrease (7.9 mm vs 6.4 mm (*P* < .01), respectively) when the resection was not done. In multivariate analysis, CA 19–9 < 500 U/mL (*P* < .01), and liver DW-MRI prescription (*P* < .01) favoured the resection.

**Conclusions:**

Due to changes in our therapeutic strategies, the NRR did not decrease during two decades despite imaging improvement.

## Background

When a solid pancreatic mass is detected, a triple-phase thoraco-abdominal computerized tomography (CT) scan has to be performed to precisely locate and measure the tumor, assess arterial and venous involvement, and detect distant visceral metastasis [1]. Improvement of CT performance could also help in the differential diagnosis of periampullary neoplasms [2] but does not replace endoscopic ultrasound (EUS) which completes this imaging work up by obtaining tissue samples via fine needle aspiration. In cases of pancreatic ductal adenocarcinoma (PDAC), pancreatectomy (with or without induction treatment depending on the tumor stage) is the pivotal point of curative treatment. However, in the late 90s, non-resection during laparotomy for pancreatectomy was common due to intraoperative findings (i.e. local invasion of major vasculature, carcinomatosis, and liver metastasis) [3].

During the last two decades, the performance of CT scan and liver diffusion weighted magnetic resonance imaging (DW-MRI) [4, 5] have improved dramatically; consequently, the non-resection rate (NRR) is expected to decrease during this period because these improvements should make it possible to detect extrapancreatic lesions (especially liver metastases) of smaller sizes. However, at the same time, two crucial points might have counterbalanced the improved efficiency of preoperative imaging. First, positive frozen sections of para-aortic lymph nodes (PALNs) were recognized by several teams, including ours, as a “new” intraoperative contraindication to surgery in patients with PDAC [6, 7]. Second, pancreatic surgeons push over the limits of resection with complex vascular reconstruction [8]; thus, exploratory laparotomy is frequently performed in locally advanced disease that finally results in more cases of non-resection according to intraoperative findings.

The present study sought to determine if the improvement in preoperative imaging over the past two decades have permitted a significant reduction in NRR among patients with PDAC eligible for pancreatectomy.

## Methods

### Patient selection

From February 1, 2000 to August 31, 2019, 751 consecutive patients with PDAC were considered eligible for intention-to-cure pancreatectomy after a multidisciplinary staff decision, and entered the operating room at Institut Paoli-Calmettes, Marseille, France. Data were entered anonymously prospectively into a clinical database (NCT02871336) and analyzed retrospectively. The study protocol was approved by the institutional review board of our hospital. No ethic approval / consent to participate was required for this retrospective observational anonymous series.

### Initial staging and decided strategy

The initial staging consisted of a physical examination, thoraco-abdominal CT scan, and CA 19–9 serum level determination (after resolution of jaundice). In April 2011, our institution acquired a dual energy spectral CT scanner that was consequently a landmark between first and second generation CT scanners and also marked the inclusion of liver DW-MRI in the imaging workup of patients. Thus, we considered two periods of inclusion: period #1 (February 2000–March 2011) and period #2 (April 2011–August 2019). As the relevance of positron emission tomography is still under debate [9], it was only performed among patients included in clinical trials. Exploratory laparoscopy was not routinely performed at initial staging or preoperatively. Surgery was decided on by a multidisciplinary team according to patients’ performance statuses, imaging, and response to neoadjuvant treatment among cases of locally advanced tumor. Vascular invasion, carcinomatosis, and liver metastasis were never confirmed based on the findings of EUS but via imaging. Exploratory laparoscopy may have been performed prior to laparotomy among cases of suspected unproven metastatic disease in order not to exclude the patient from a potentially curative strategy. Laparoscopy was performed only to detect carcinomatosis or liver metastasis, and not to determine local vascular invasion. Consequently, all patients included in the present study had a negative exploratory laparoscopy if performed.

### Surgery

Pancreatectomy was performed via a subcostal incision or laparoscopic approach according to the tumor site and surgeon preference. First, a thorough abdominal exploration was performed. Contraindications for resection were intraoperative histologically proven carcinomatosis, liver metastasis, or PALNs metastasis [7]. Invasion of the superior mesenteric artery, celiac axis, or hepatic artery were not considered as contraindications to resection in highly selected cases (fit patients, objective response to induction treatment, long [> 6 months] follow up without any metastasis detected, low (< 500 IU/mL) [10] CA 19–9 serum level, and no neoadjuvant chemoradiation). Venous resection was performed as described previously [8]. Specimens were inked to facilitate margin assessment according to period of inclusion [11]. Adjuvant chemotherapy was administered to fit patients according to a multidisciplinary team decision.

### Study parameters

Several variables were evaluated: age, sex, body mass index (BMI), CA 19–9 serum level (U/mL; at diagnosis and after jaundice resolution), biliary stenting, neoadjuvant treatment administration, delay (days) from CT scan to surgery, period of staging (i.e. period #1 or period #2), liver DW-MRI and positron emission tomography request, and exploratory laparoscopy prior to intention-to-treat surgery (with calculation of the median delay (days) between laparoscopy and surgery). In cases of intraoperative contraindication, the etiology (i.e. carcinomatosis, liver metastasis, vascular involvement, or PALNs invasion) and size (mm) of the largest extra pancreatic tumor were noted. Type of surgery (i.e. pancreaticoduodenectomy (PD), total pancreatectomy, or distal pancreatectomy), venous, and/or arterial resections, postoperative courses, pathologic findings (R1 resection was defined by tumor cells within 1 mm to resection margin), and adjuvant treatment administration were also noted. Survivals were calculated from date of diagnosis until the date of death or the censor date (December 2019) for living patients.

### Statistical analysis

Categorical variables were compared using Fisher’s exact test or the chi-squared test, and continuous variables using the student’s *t*-test. Multivariate analysis was performed using stepwise logistic regression by integrating factors identified in the univariate analysis with *P* < .1. Survival analysis were performed according to the Kaplan-Meier method; survival curves were compared using the Wilcoxon test. Data analyses were performed using GraphPad Prism version 6 (GraphPad Software Inc., La Jolla, CA, USA) and SPSS® version 24 (SPSS Inc., Chicago, IL, USA). Analyses items with *P* < .05 were considered statistically significant.

## Results

The characteristics of the 751 patients are summarized in Table 1. All patients underwent a preoperative CT scan with a median delay to surgery of 18 days (range 1–55). Liver DW-MRI was performed among 407 patients (54%). A neoadjuvant treatment was delivered in 337 patients (45%). Pancreaticoduodenectomy was the main type of surgery (51%); a venous resection was achieved in 159 patients (21%). According to the intraoperative findings, the NRR was 24.5% (184 patients). Causes for non-resection were vascular invasion (10%), liver metastasis (7%), carcinomatosis (5%), and PALNs invasion (3%). Among the patients who underwent a negative explorative laparoscopy (*n* = 82) and who entered the operating room, 23 (28%) finally where not resected due to a contraindication founded during laparotomy (median delay from laparoscopy to surgery: 18 days (range: 5–37)). In patients who underwent resection (*n* = 567), overall morbidity and 30-days mortality rates were 49 and 4.2% respectively. Three hundred and fifty-two patients (62%) received an adjuvant treatment; the median overall survival rate was 36 months.

**Table 1:** Characteristics of the 751 patients who entered the operating room

Sex Ratio (M/F)	1.05 (384/367)
**Median Age** (range)	67 (25–86)
**Mean BMI** (±SD)	24.7 (±4.35)
**Period** (%)	
1 (February 2000–March 2011)	224 (30)
2 (April 2011–August 2019)	527 (70)
**Biliary Stenting** (%)	418 (58%)
**Work up Imaging** (%)	
CT-scan	751 (100)
Liver Magnetic Resonance Imaging	407 (54)
Positron Emission Tomography	69 (9)
**Median delay CT-Surgery** (days) (range)	18 (1–55)
Mean CA 19–9 serum level* (UI) (±SD) (after jaundice resolution)	552 (±1200)
**Neoadjuvant Treatment** (%)	337 (45)
**Explorative Laparoscopy** (%)	82 (11)
**Type of Surgery** (%)	
Exploration without Resection	184 (25)
Pancreaticoduodenectomy	384 (51)
Distal Pancreatectomy	146 (19)
Total Pancreatectomy	37 (5)
Venous Resection	159 (21)
Arterial Resection	19 (2.4)
En-bloc Resection of Neighbours Organs	43 (5.7)
**Reason of Non-resection** (%)	
Carcinomatosis	35 (5)
Liver Metastasis	49 (7)
Vascular Invasion	75 (10)
Distant Lymph Node Invasion	25 (3)
Mean Size of Extra Pancreatic Metastasis (mm) (±SD)	7.5 (±4.6)
**Morbidity** (%)	
Resected patients (Overall / Grades 3 to 5 Clavien-Dindo)	279 (49) / 85 (15)
Hemorrhage	47 (8)
Clinically Revelant Postoperative Pancreatic Fistula	108 (219
Reintervention	43 (8)
Non-resected patients (Overall / Grades 3 to 5 Clavien-Dindo)	33 (1.8) / 6 (3.2)
**30- / 90-days Mortality** (%)	
Resected patients	24 (4.2) / 30 (5.3)
Non-resected patients	4 (2.2) / 10 (5.4)
**Pathologic Findings** (Resected patients)	
T1/2 (%)	205 (36)
T3/4 (%)	362 (64)
Median Number of Lymph Nodes (range)	13 (2–44)
N+ (%)	329 (58)
R1 (%)	258 (46)
Perineural Invasion (%)	385 (68)
**Adjuvant Treatment** (%)(Resected patients)	352 (62)

(*BMI* Body Mass Index; *SD* Standard Deviation, * at diagnosis)

### Period #1 versus period #2 (Table 2)

**Fig. 1:**
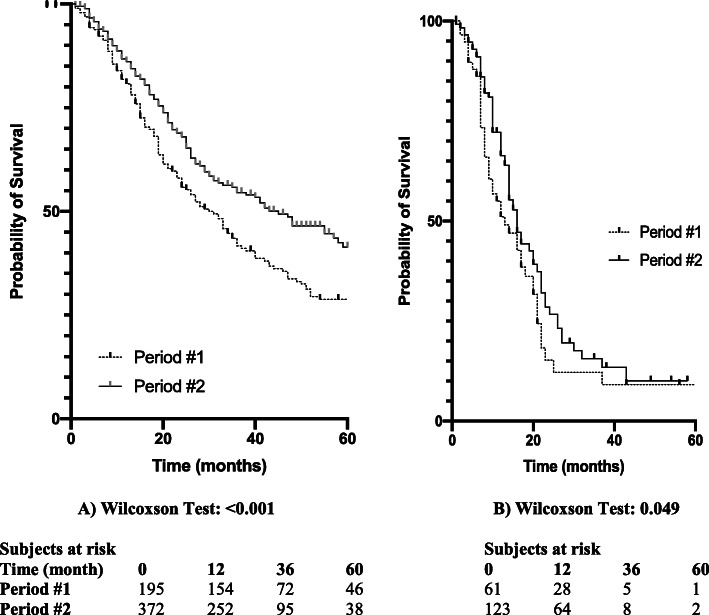
Survival in patients who benefit (1**a**) or not (1**b**) from resection according to period of surgery

More patients underwent surgery during period #2 (224 versus (vs) 527; *P* < .001). Median delay between CT and surgery decreased (21 days to 16 days, *P* < .01), and liver DW-MRI was significantly most prescribed during period #2 (14% vs 75%, *P* < .01). While vascular invasion (13% vs 8%), liver metastasis (5% vs 7%), carcinomatosis (6% vs 4%) rates remained stable, PALNs invasion rate (0.4% vs 4.6%; *P* < .001) significantly increased over the 2 periods. In patients who underwent resection, postoperative courses were comparable for most criteria excepted for the 90-days mortality rate that decreased from 8.2 to 3.8% (*P* = .03), and for adjuvant treatment administration rate that increased from 47 to 70% (*P* < .001). Pathologic findings showed significantly more lymph node harvested (median number: 10 vs 15), and more lymph node invasion (51% vs 62%) during period #2 when comparing with period #1. Median overall survival time was higher during period#2 if patients benefit (30 vs 45 months; *P* < .01) (Fig. 1a) or not from resection (13 vs 16 months; *P* = .049) (Fig. 1b).

**Table 2:** Characteristics of the 751 patients who entered the operating room according to period of workup imaging

	Period 1	Period 2	***P*** value
**n**	256	495	**< 0.01**
**Sex Ratio** (M/F)	1.11	1.02	0.58
**Median Age** (range)	67 (32–85)	65 (25–86)	0.19
**Mean BMI** (±SD)	25.3 (±4.64)	24.4 (±4.1)	**0.01**
**Biliary Stenting** (%)	118 (46)	300 (61)	**< 0.001**
**Work up Imaging** (%)			
CT-scan	224 (100)	527 (100)	1
Liver Magnetic Resonance Imaging	35 (14)	372 (75)	**< 0.001**
Positron Emission Tomography	7 (2.7)	62 (13)	**< 0.001**
**Median delay CT-Surgery** (days) (range)	21 (1–55)	16 (1–48)	**< 0.001**
Mean CA 19–9 serum level* (UI) (±SD) (after jaundice resolution)	552 (±937)	552 (±1275)	1
**Explorative Laparoscopy (%)**	23 (9)	59 (12)	0.27
**Neoadjuvant Treatment** (%)	118 (46)	219 (44)	0.64
**Type of Surgery (%)**			
Exploration without Resection	61 (24)	123 (25)	0.79
Pancreaticoduodenectomy	152 (59)	232 (47)	**0.001**
Distal Pancreatectomy	35 (14)	111 (22)	**0.005**
Total Pancreatectomy	8 (3)	29 (6)	0.11
Venous Resection	36 (14)	123 (25)	**< 0.001**
Arterial Resection	0	19 (4)	**< 0.001**
En-bloc Resection of Neighbours Organs	16 (6)	27 (5)	0.74
**Reason of Non-resection (%)**			
Carcinomatosis	15 (6)	20 (4)	0.28
Liver Metastasis	12 (5)	37 (7)	0.16
Vascular Invasion	33 (13)	42 (8)	0.07
Total	60 (23)	99 (20)	0.3
Distant Lymph Node Invasion	1 (0.4)	24 (5)	**< 0.001**
Mean Size of Extra Pancreatic Metastasis (mm) (±SD)	7.9 (±5.2)	6.4 (±3.9)	**< 0.01**
**Morbidity** (%)(resected patients)	195	372	
Overall / Grades 3 to 5	93 (48) / 28 (14)	186 (35) / 57 (15)	0.66 / 0.3
Hemorrhage	22 (11)	25 (11)	0.08
Clinically Revelant Postoperative Pancreatic Fistula	37 (19)	71 (13)	0.99
Reintervention	18 (9)	25 (4.7)	0.31
**30- / 90-days Mortality** (%)(resected patients)	11 (5.6) / 16 (8.2)	13 (3.5) / 14 (3.8)	0.27 / **0.03**
**Pathologic Findings** (resected patients)			
T1/2 (%)	71 (36)	134 (36)	0.74
T3/4 (%)	124 (64)	238 (64)	0.93
Median Number of Examined Lymph Nodes (range)	10 (2–23)	15 (4–44)	**< 0.01**
N+ (%)	99 (51)	230 (62)	**0.01**
R1 (%)	81 (42)	177 (48)	0.18
Perineural Invasion (%)	122 (63)	263 (71)	0.06
**Adjuvant Treatment** (%)(resected patients)	91 (47)	261 (70)	**< 0.001**

(*BMI* Body Mass Index; *SD* Standard Deviation, * at diagnosis)

**Table 3:** Characteristics of the 751 patients who entered the operating room according to resection achievement

	Non Resected	Resected	***P uni.***	***P multi (95%CI)***
**n (%)**	184 (25)	567 (75)	–	–
**Sex Ratio** (M/F)	1.45	1.09	0.08	–
**Median Age** (range)	66 (35–79)	65 (26–86)	0.38	–
**Mean BMI** (±SD)	24.3 (±4.2)	24.7 (±4.67)	0.16	–
**Period** (%)			0.51	–
1 (February 2000–March 2011)	56 (25)	168 (75)		
2 (April 2011–August 2019)	128 (24)	399 (76)		
**Work up Imaging** (%)				
CT-scan	184 (100)	567 (100)	1	–
Liver Magnetic Resonance Imaging	73 (40)	334 (59)	**< 0.001**	**< 0.01 (3.34 [2.24; 5.01])**
Positron Emission Tomography	20 (11)	49 (9)	0.38	–
**Median delay CT-Surgery** (days) (range)	18 (6–55)	18 (1–43)	0.29	**–**
**Mean CA 19–9 serum level** (UI) (±SD)	848 (±1004)	391(±852)	**< 0.01**	< 0.01 (1.99 [1.21; 3.71])*
**Explorative Laparoscopy (%)**	21 (11)	61 (11)	0.79	–
**Neoadjuvant Treatment** (%)	70 (38)	267 (47)	**0.033**	0.17 (1.12 [0.91; 1.71])

(* in patients with CA 19–9 < 500UI/mL)

### Non-resection rate

NRR were comparable between periods #1 and #2 (25% vs 24%, respectively), as well as if we focused in patients with resectable disease (i.e. who did not receive neoadjuvant treatment) at diagnosis (13% vs 18% (*P* = .07), respectively). Carcinomatosis, liver metastasis, and local vascular contraindication rates were stable when comparing periods #1 and #2 whereas PALNs invasion contraindication rate increased (0.3% vs 5% (*P* < .001), respectively); the mean size of the bigger extra pancreatic tumor significantly decrease (7.9 mm vs 6.4 mm (*P* < .01), respectively) when the resection was not done. In multivariate analysis, CA 19–9 < 500 U/mL (*P* < .01), and liver DW-MRI prescription (*P* < .01) favoured the resection (Table 3). When a liver DW-MRI was not achieved, the relative risk of non-resection was 1.8 (95% CI [1.43; 2.72].

## Discussion

Our study revealed that the NRR did not decrease during the last 2 decades among patients with PDAC entering the operating room for an intention-to-cure pancreatectomy. The observed overall NRR of 24.5% was consistent with those of previously reported series among patients diagnosed with PDAC [12].

### Staging

There is no doubt that the technical performances of CT and DW-MRI have improved over the last 2 decades. As a surrogate of this enhancement, the sizes of the extra-pancreatic disease which contraindicate the resection significantly decreased in the second period. Moreover, vascular contact is certainly better assessed nowadays via imaging eventually associated to EUS [13], but these improvements were not correlated with a reduction in NRR, which was surprising. It is now well-known that a short (< 30 days) delay between CT scan and surgery is crucial in order not to increase the NNR [14]. In our series, the majority of patients underwent a 3-phase CT scan with an “optimal” (18 days) delay [15–17] prior to surgery whatever the period of inclusion. This insured a relevant comparison between the two periods and the absence of NRR reduction could not be attributed to a higher delay between imaging and surgery in period #1 that counterbalanced the enhancement of imaging.

Routine laparoscopy was not performed, and this could be a drawback of our study; we suppose that any included patients could have been spared exploration if laparoscopy was performed. However, laparoscopy is not routinely performed worldwide and a recent large series reported that about 10% of patients underwent this procedure [18]. Similarly, its relevance remains under debate [19]. Nevertheless, we wanted to highlight that 12% of patients in our series who underwent an exploratory laparoscopy were finally found to have a contraindication to resection intraoperatively (with a “short” delay from laparoscopy to exploration of 18 days). This suggested that negative exploratory laparoscopy did not systematically imply a resection, mainly because it is difficult to explore major vasculature involvement in this way. As we strongly believe that positive PALNs are a contraindication to resection in patients with PDAC [7], we started a prospective evaluation of routine laparoscopy at diagnosis with PALN resection this year. This strategy will help assess the laparoscopic relevance at diagnosis, and we will report our results after the first 100 cases have been assessed.

Finally, the CA 19–9 serum level was independently associated with a higher NRR, reminding us the importance of the biological dimension in patients with PDAC [20, 21].

### Non-resection rate

Not surprisingly, liver DW-MRI appeared to be a crucial tool for staging as already reported [4, 22]. However, it could not be considered in isolation. Indeed, liver DW-MRI is a “focal” exam that only screens a specific zone (the liver and pancreas), has blind spots (subcapsular small liver metastasis and interference of duct dilation in case of bile duct obstruction) and consequently could not replace CT scan. To reinforce this, we showed that a CT/DW-MRI combination significantly reduced the NRR compared with patients who only underwent a CT scan (RR, 1.8). However, we did not observe a decrease in NRR between the two periods, despite a significantly higher liver MRI rate during period #2 among patients who did not have locally advanced disease at diagnosis. This could be a contradictory observation; however, two major changes in the patients’ strategies dramatically impacted period #2 at our institution and could explain the lack of reduction in NRR. First, we integrated the intraoperative PALNs assessment in our decision-making strategies for patients with PDAC scheduled for pancreatectomy [6]. Indeed, among cases with positive frozen section results, we did not perform tumor resection [7]. Thus, not surprisingly, we observed a significantly higher NRR rate due to PALNs invasion during period #2. However, this relevant difference was not sufficient to increase the overall NRR probably because very few patients presented with PALNs invasion (3%). Second, since 2010, pancreatic surgery was performed by a dedicated surgical team [8]. This increased both the number of cases and the complexity of the procedures performed. Consequently, we will have decreased the risk of achieving non-resection if our “local” criteria of resection had remained constant between the two periods. However, we pushed over the limits of resectability with venous and arterial resection [8] and this increased the risk of non-resection resulting in the absence of significant impact on the NRR.

Finally, we did not observe a reduction in NRR when considering patients with resectable disease at diagnosis. However, as the difference was not significant (due to an insufficient sample size), we noted a trend of NRR reduction between the two periods in this sub-population (13% vs 18%; *P* = .07). By suppressing the potential bias due to local invasion, we could argue that CT/DW-MRI helped to better identify liver metastasis or carcinomatosis at staging among patients with resectable disease.

Our study was not designed to assess oncology outcomes. However, we observed encouraging changes in survival between the two periods, probably due to improvements in surgery and perioperative treatment.

### Perspectives

Our study supported the notion that patients diagnosed with pancreatic PDAC must benefit from CT and liver DW-MRI. Inclusion of the CA 19–9 level and laparoscopy might also help to reduce the NRR and consequently spare the patient from useless exploratory surgery. In the future, imaging [23–25] development in association with EUS [26, 27], and assistance during laparoscopy [28] could improve the relevance of tumor staging. However, the aim of staging is to detect existing distant metastasis or vascular invasion that precludes resection. This concept will probably be challenged in the near future by new biomarkers such as circulating tumor cell number [29] or genomic assessment of the tumor [30, 31] that could predict poor outcomes. If such staging became obvious and relevant, the pancreatic surgeon will then face a complex ethical situation: is a patient without any contraindication to resection based on the “classic” imaging staging to be spared resection because these new tools predict rapid disease progression?

## Conclusion

Due to changes in our therapeutic strategies, the NRR did not decrease these last two decades despite imaging improvement. However, our study highlighted the crucial role of combining CT and DW-MRI to spare patients from surgical exploration.

## Data Availability

Data are available upon request from the corresponding author.

## Abbreviations

CT: Computerized tomography
EUS: Endoscopic ultrasound
PDAC: Pancreatic ductal adenocarcinoma
DW-MRI: Fusion weighted magnetic resonance imaging
NNR: Non-resection rate
PALN: Para-aortic lymph node
